# The need for a system view to regulate artificial intelligence/machine learning-based software as medical device

**DOI:** 10.1038/s41746-020-0262-2

**Published:** 2020-04-07

**Authors:** Sara Gerke, Boris Babic, Theodoros Evgeniou, I. Glenn Cohen

**Affiliations:** 1 Project on Precision Medicine, Artificial Intelligence, and the Law; Petrie-Flom Center for Health Law Policy, Biotechnology, and Bioethics at Harvard Law School, Harvard University, Cambridge, MA USA; 2 INSEAD, 1 Ayer Rajah Ave, Singapore, 138676 Singapore; 3 INSEAD, Boulevard de Constance, 77300 Fontainebleau, France; 4 Harvard Law School; Petrie-Flom Center for Health Law Policy, Biotechnology, and Bioethics at Harvard Law School, Harvard University, Cambridge, MA USA

**Keywords:** Health policy, Law

## Abstract

Artificial intelligence (AI) and Machine learning (ML) systems in medicine are poised to significantly improve health care, for example, by offering earlier diagnoses of diseases or recommending optimally individualized treatment plans. However, the emergence of AI/ML in medicine also creates challenges, which regulators must pay attention to. Which medical AI/ML-based products should be reviewed by regulators? What evidence should be required to permit marketing for AI/ML-based software as a medical device (SaMD)? How can we ensure the safety and effectiveness of AI/ML-based SaMD that may change over time as they are applied to new data? The U.S. Food and Drug Administration (FDA), for example, has recently proposed a discussion paper to address some of these issues. But it misses an important point: we argue that regulators like the FDA need to widen their scope from evaluating medical AI/ML-based products to assessing systems. This shift in perspective—from a product view to a system view—is central to maximizing the safety and efficacy of AI/ML in health care, but it also poses significant challenges for agencies like the FDA who are used to regulating products, not systems. We offer several suggestions for regulators to make this challenging but important transition.

## Introduction

Artificial intelligence (AI), particularly its subset Machine learning (ML), has the potential to improve health care systems worldwide, for example, by optimizing workflows in hospitals, providing more accurate diagnoses, and bringing better medical treatments to patients. However, medical AI/ML also creates new challenges that we, as a society, and especially current regulators like the U.S. Food and Drug Administration (FDA), need to face: Which medical AI/ML-based products should undergo review by regulators? What evidence should regulators require makers of AI/ML-based software as a medical device (SaMD) to submit as a prerequisite to permit marketing? How to ensure the improvement of AI/ML-based SaMD’s performance in real-time while safeguarding their safety and effectiveness?

Some AI/ML-based SaMD have already received marketing authorization in the U.S., including IDx-DR in 2018, the first AI/ML diagnostic that provides a screening decision for the eye disease diabetic retinopathy, which its maker claims is the “first ever autonomous AI system cleared by the FDA to provide a diagnostic decision^1,2^”. Many patients and physicians are particularly concerned about such “autonomous” devices. The current AI/ML-based SaMD that received marketing authorization by the FDA have what the FDA has called “locked” algorithms—they do not evolve over time and do not use new data to alter their performance^3^. If the algorithm changes through usage, such SaMD will, at present, likely require another FDA round of review^3^. Thus, AI/ML makers will probably be inclined not to update their SaMD—both to reduce the cost and effort, but also because there are risks to proposing an update (and thus signaling a deficiency of the baseline product) if FDA does not approve the update or there is a significant delay^4^. For these reasons, the most valuable asset of AI/ML, its ability to improve by learning from data, may not be fully harnessed.

Elsewhere we have discussed the FDA’s recent attempt to wrestle with what we call “the update problem”, its treatment of “locked” versus “adaptive” algorithms, and have made proposals for how the FDA’s approach might be improved, such as through continuous monitoring processes that consider specific risks of AI/ML systems^4^. In this article, we make a more fundamental point: The FDA and its sister regulators in other countries have primarily been product regulators—they review and ultimately approve or reject medical products such as drugs and devices. AI/ML-based SaMD and perhaps other future ways in which AI/ML will be incorporated in medical products will require the agency and its sister regulators to shift more towards a “system” view. Even IDx-DR, the most “autonomous” AI/ML-based product authorized for marketing by the FDA, is not an island. It is one part of a larger system involving various kinds of human involvement—from health care teams inputting the data to physicians reacting to the AI recommendation to insurers deciding whether to reimburse only for certain courses of action. It is the entire system that must be evaluated—a concept that we call the “system approach”. We discuss how an agency like the FDA should think about AI/ML in a systemic way and how this puts pressure, for example, on traditional concepts of the limits of the FDA’s jurisdiction—in particular, the current approach that the FDA does not regulate the practice of medicine^5^. While a full-scale move into the system approach is currently infeasible for regulators, we discuss how they might take further steps in this direction, which can improve the public’s confidence in the use of AI in health care.

## What are AI/ML-based SaMD

Several medical AI/ML-based products must undergo review by regulators. For example, in the U.S., a medical device is defined in Section 201(h) of the Federal Food, Drug, and Cosmetic Act and is “an instrument, apparatus, implement, machine, contrivance, implant, in vitro reagent, or other similar or related article, including any component, part, or accessory, which is (…) intended for use in the diagnosis of disease or other conditions, or in the cure, mitigation, treatment, or prevention of disease (…), which does not achieve its primary intended purposes through chemical action within or on the body of man (…), and which is not dependent upon being metabolized for the achievement of its primary intended purposes”.

Under U.S. law, some AI/ML-based software functions may not fall under the device definition (e.g., certain clinical decision support software under Section 520(o)(1)(E) of the Federal Food, Drug, and Cosmetic Act), but some do. In particular, the term “Software as a Medical Device” (SaMD) is used to refer to software that is on its own a medical device, “without being part of a hardware medical device”^6,7^. While not everyone is supportive of the SaMD construct, given that the FDA has committed to it, in what follows we examine how it can be enriched by going beyond a pure product worldview.

## Why the product worldview is inadequate for AI/ML-based SaMD

AI/ML-based SaMD raise new challenges for regulators. As compared to typical drugs and medical devices, we argue that due to their systemic aspects, AI/ML-based SaMD will present more variance between performance in the artificial testing environment and in actual practice settings, and thus potentially more risks and less certainty over their benefits. Variance can increase due to human factors or the complexity of these systems and how they interact with their environment. Unlike drugs, the usage of software and generally Information Technologies (IT) is known to be highly affected by organizational factors such as resources, staffing, skills, training, culture, workflow, and processes (e.g., regarding data quality management)^8^. There is no reason to expect that the adoption and impact of AI/ML-based SaMD will be consistent, or even improve performance, across all settings. A good cautionary tale comes from the use of computer-aided detection (CAD) for mammography, which was, in particular, financially encouraged by the Centers for Medicare and Medicaid Services in the early 2000s as a way to improve breast cancer detection. As a study in JAMA Internal Medicine showed, because of the way physicians interacted with CAD they performed no better (and in some ways worse) when CAD was introduced^9^. Human judgment also introduces well-known biases into an AI/ML environment, including, for example, inability to reason with probabilities provided by AI/ML systems, over extrapolation from small samples, identification of false patterns from noise, and undue risk aversion^10^. Even with a single user in a single setting, there may be poor consistency: over time—both in terms of experience but even in the course of the day—we may see more risk aversion or alert fatigue of physicians. Judges, for example, have been documented to have a different tolerance for risk during the course of a single day^11^. These make it much more difficult for regulators to decide whether permitting marketing authorization is warranted, but also, for example, for a purchaser of an AI/ML system to determine whether it will add value to that individual practice or hospital. Regulators like the FDA have already started considering some of these issues, such as requiring training programs and human factors validation testing^12^. However, there are more nuanced and possibly complex systemic issues to consider.

AI/ML-based SaMD also differ from other medical technologies, such as the da Vinci surgical system^13^, because (1) they have the capacity to continuously learn, (2) they have the potential to become ubiquitous in medical interactions and make recommendations (unlike robotic-assisted surgical systems), and (3) the way they reach their recommendations is often opaque to physicians. For example, the latter factor creates particular puzzles for regulators: Do different physicians interact differently with the same algorithm if they believe the basis for its decision-making is explainable (even if they do not themselves understand the basis for that decision) as opposed to when they believe the algorithm is more opaque?

Variance will likely further increase as future AI/ML-based SaMD begin to interact with physicians dynamically, for example, by responding to the physician’s manipulations and possibly also becoming better attuned to the preferences of each individual user. The more human-AI interactions in decision-making, the more uncertainty as to what outcomes the AI/ML-based SaMD (and similar medical devices) will actually produce in clinical settings due to factors outlined above. Thus, even attempts to engage in human factors testing will have difficulties determining outcomes if evaluation is not done in actual practice settings since outcomes are likely to vary much more than, say, the use of a drug on a particular type of patient.

These insights are well captured by the so-called Kasparov’s Law, named for the chess player Garry Kasparov: the idea that a weak human cooperating with a machine under a good process is superior to a strong computer alone and, surprisingly, to a strong human with a machine under a weak process^14^. “Strong” and “weak” refer to the skill or lack thereof of the human, but the key focal point is the “process”. Kasparov made this observation during a 2005 chess tournament in which the winners were, counterintuitively, two amateur chess players who used three computers and who were able to better manipulate and coach their computers to take an in-depth look into positions than chess Grandmasters and participants with greater computational power^14^. Kasparov’s observation can also be seen in other situations in daily life. For example, most people use Google as a search platform, but some are quicker (and more efficient) in identifying the information needed by entering the “right” key words into the engine. The key insight here is that we cannot know whether the AI/ML-based SaMD will improve outcomes without knowing more about the process.

To see this in the context of health care, consider an example the FDA gives in a recent discussion paper: The FDA considers an AI/ML-based SaMD that “receives electrocardiogram, blood pressure, and pulse-oximetry signals from a primary patient monitor” and then “signals are processed and analyzed to detect patterns that occur at the onset of physiologic instability”, with an alert for the physician warning “that prompt clinical action is needed to prevent potential harm to the patient”^3^. Imagine the FDA permitted marketing of such an AI/ML-based SaMD. The FDA then asks what should happen if the company retrained its algorithm using additional data and “the revised algorithm has the same sensitivity and false-alarm rate as the previous version”, but it can now alert “15 minutes prior to the onset of physiologic instability, which the previous version of the algorithm could not do”^3^?

This seems like a great advantage, but without robust human factors testing, we cannot know if the new version is actually better. It is possible that the way humans react to these alerts going off further away in time from the signs of instability makes the algorithm less effective; for example, because the alert and the instability are no longer paired so closely in time, the users begin to doubt the probative value of the alerts and begin discounting them more often.

In addition to the uncertainty introduced by human users’ reactions to AI/ML output, the system view reveals additional complex and interacting elements to consider—interactions between different parts of the care team with the AI, the payment structure, possibly data providers, software components providers, and trainers. For example, an AI/ML-based SaMD might be approved by regulators on the understanding that a physician can always overrule its recommendations. But what happens if that, while formally true, is nonetheless in practice a fairly rare occurrence because a payer will only reimburse for that which is recommended by the AI/ML system? Should that be considered as part of the regulatory approval process?

The key insight that the health care impact of AI/ML-based SaMD depends on many factors of a broader system indicates that the regulators’ focus should be on designing an appropriate process for managing this new environment taking a more system approach than a product one. Unless regulatory review is attuned to such system aspects as outlined above, it will be woefully incomplete. The insight also helps us see that, perhaps paradoxically, more autonomous AI/ML systems can create more, rather than less, predictability in the emerging human-AI environment.

## What would it mean to fully adopt a system approach

Clearly, taking the system perspective seriously makes the job of regulators like the FDA evaluating AI/ML-based SaMD much more difficult. A full system approach would require the regulator to collect data on a myriad of information beyond its current regulatory gaze and perhaps even beyond its legal mandate, requiring additional statutory authority^3^—the reimbursement decisions of insurers, the effects of court decisions on liability, any behavioral biases in the process, data quality of any third-party providers, any (possibly proprietary) machine learning algorithms developed by third parties, and many others. In a full system approach, the regulator would then issue a limited regulatory authorization that tracks factors like the ones discussed above. Indeed, the regulator might even require approval to come at the level of a specific hospital, possibly with specific trained and authorized users, including, among other things, detailed hospital-level information about how the AI/ML-based SaMD is integrated into the workflow and staffing levels of that hospital, how the practice style and training of the physicians at that hospital interact with it, how the payers in that market authorize or do not authorize reimbursement for actions that deviate from its recommendations, and how the tort law in that jurisdiction intersects with provider decision-making.

This would be a huge change from what the FDA and its peer regulators in other countries currently consider as part of their review. One could, perhaps, find some loose analogies in the way the United Kingdom’s specialized Human Fertilisation and Embryology Authority (HFEA) licenses individual clinics for particular reproductive technology uses such as maternal spindle transfer (MST) or pronuclear transfer (PNT), two mitochondrial replacement techniques to prevent the transmission of serious mitochondrial disease from a mother to her infant^15^. To act lawfully, clinics need to get a license from the HFEA to carry out one or both of these techniques—they need to show the capability to perform MST and/or PNT^15^. In addition, they also must receive another approval from the HFEA when using one of these techniques for a particular patient^15^. Something similar could, in theory, be done for AI/ML-based SaMD, but the burden on the regulator would be much more demanding since in making such local evaluations it must consider far more facets of health care delivery, insurance, and law, and do so for a much wider set of technologies. Such an approach would also raise difficult questions about how to far upstream regulators would need to go, for example, in validating “golden datasets” as ground truth comparators.

Moreover, because the system itself may change, even if the AI/ML remains “locked”, a full system approach would not treat premarket approval even at the level of a hospital as a “one and done”, but instead tentative and subject to reevaluation. To be sure, the FDA currently monitors the safety of medical products through its Sentinel program, and in September 2019, the FDA announced its goal to enlarge Sentinel to three distinct coordinating centers with more monitoring capabilities^16,17^. But a full system approach would require far more than this. Finally, a full system approach would require the FDA at least to take some steps in contravention of one of its shibboleths: that the FDA does not regulate the practice of medicine^5^. Such multi-faceted changes in the processes and possibly mandates of the regulators may be necessary in the world of increasingly sophisticated AI/ML systems, but a full change may not be possible or even desirable as it may limit innovation or negatively affect the behavior of stakeholders.

## Transitioning from a product to a system approach: first steps

We believe that a full system approval and monitoring approach is out of reach for today’s regulators, especially because of expertise, resources, political obstacles, and the difficulties in controlling human behavior^18^. The Perfect, however, must not be the enemy of the Good. If regulators cannot realistically take the full system approach, they can at least somewhat widen their perspective. Doing so is also in harmony with the FDA’s increasing emphasis in its AI/ML-based SaMD publications of implementing more “real-world performance monitoring”^3,19^.

Regulators like the FDA can, for example, demand fuller human factors analysis of how actual physicians and others of the health care team such as nurses react to outputs of particular AIs and require training for users to help minimize variance. As it stands, the FDA does not regulate the practice of medicine and thus does not oversee the training of medical professionals. However, even with those restrictions in place, it can require the AI maker to set up a training program for their product, such as in the case of IDx-DR where the FDA required a training program including instructions on how to acquire and process quality images^12^. Regulators could require more, such as ongoing system monitoring, periodic retraining, software and usage inspections, review of aggregate usage statistics (e.g., to identify possible drifts in treatment frequencies and decision styles of users)^4^. They could also demand data and model validation and robustness analysis (e.g., via multiple re-trainings with different data subsets and data perturbations) of the AI/ML, such as due to data quality or adversarial attack issues^4^. Further, regulators could also require testing variants that provide humans with different degrees of freedom: For example, users’ discretion can be more or less limited in cases where devices provide probabilistic recommendations such as IDx-DR; or the AI/ML-based SaMD may provide more or fewer alternative recommended courses of action or even usage parameter choices. Regulators may also simply require clinical trials of the AI/ML-based SaMD as used in actual planned clinical settings. Moreover, regulators could also request data collected outside traditional clinical trials such as from Fitbits and other wearables capturing users’ behavioral changes over time as well as electronic health records capturing all decisions that may be related to the use of an AI/ML-based SaMD^20^.

In a sense, it may be useful to reframe what the hospitals and practices are doing: they are not merely buying an AI/ML-based tool, but hiring one. Cognitive testing of a physician will not tell you how they will do when added to a preexisting team in a particular health system, and employees need to be continuously assessed. The same is true for AI/ML-based SaMD.

## The system approach and the special case of “Locked” versus “Adaptive” algorithms

All AI/ML-based SaMD that the FDA has thus far reviewed have been cleared or approved as “locked” algorithms, which it defines as “an algorithm that provides the same result each time the same input is applied to it and does not change with use”^3^. The agency is currently developing a strategy for how to regulate “unlocked” or “adaptive” AI/ML algorithms—algorithms that may change as they are applied to new data^3^.

This is a welcome development. Much of the value of medical AI/ML-based SaMD is in their ability to update as new and hopefully more representative data, for example, becomes available. Unfortunately, the task of reviewing such an “update” is much more difficult when we consider the system rather than the product perspective.

Elsewhere we have suggested that regulators should prioritize risk monitoring to address the “update problem”^4^. We articulated some key features that risk monitoring should focus on (i.e., concept drift, covariate shift, and instability) and suggested some ways to implement it^4^. Our goal is to emphasize that the tasks adopted by a true system view for regulating adaptive AI/ML-based SaMD are indeed even more demanding since they consider many more facets of the delivery of care. If there were no human involvement, for a hypothetically truly fully autonomous AI (despite the company’s marketing, the first AI/ML diagnostic IDx-DR is not), the update could be approved based on retesting a reference set of patients, all prior patients, or even on simulated patient data. But when humans are involved, the system perspective requires considering how the update interacts with human usage and organizational factors. To be sure, in some instances, AI/ML-based SaMD updates will have no effect or improve things from a human factor perspective. The point is that determining the effects of the update is much more challenging for a regulator when there is significant human involvement in decision-making.

## Conclusion

AI/ML-based SaMD pose new safety challenges for regulators. They need to make a difficult choice: either largely ignore systemic and human factor issues with each approval and subsequent update or require the maker to conduct significant organizational and human factors validation testing with each update resulting in increased cost and time, which may, in turn, chill the desire of the maker to engage in potentially very beneficial innovations or possible updates. Striking the right balance is a challenge that may take time to resolve. However, ignoring all systemic aspects of AI/ML-based SaMD, such as those we outlined, may not be an option.
